# Acellular placental extract outcomes in post-traumatic osteoarthritis model

**DOI:** 10.1016/j.ocarto.2025.100729

**Published:** 2025-12-12

**Authors:** Xue Ma, Marcel G. Brown, Matthew S. Gwilt, Kaitlin Henry, Ayobami Ogunsola, Jesse Champi, Seth Tomblyn, Scott Washburn, John S. Shields, Thomas L. Smith

**Affiliations:** a Department of Orthopaedic Surgery, Wake Forest University School of Medicine, Winston-Salem, NC, 27157, United States; b Wake Forest University School of Medicine, Winston-Salem, NC, 27157, United States; c Plakous Therapeutics, Inc., Winston-Salem, NC, 27103, United States

**Keywords:** Post-traumatic osteoarthritis (PTOA), Osteoarthritis, Cartilage, Human placental extract (HPE)

## Abstract

**Objective:**

Evaluate the effect of human placental extract (HPE) on healthy and osteoarthritic articular cartilage.

**Method:**

Post-traumatic osteoarthritis (PTOA) rodent model was utilized to assess functional and histologic outcomes of HPE administration at 1, 2 and 3 months. Matrix metalloproteinase (MMP), glycosaminoglycan (GAG) content were assessed via human and porcine cartilage explants exposed to either HPE or saline cohorts.

**Results:**

Improved gait function of PTOA-induced rodents treated with HPE in 5 measured parameters, persisting to the 3-month time point against the saline control. No significant difference in medial or lateral joint space was found on MicroCT evaluation after normalizing results to the contralateral uninjured limb. OARSI scores of the medial compartment of PTOA rodents at 3-months showed preservation of cartilage in the HPE-treated cohort with a score of 1.8 for HPE versus 9.17 for saline (*p* < 0.05). Porcine cartilage explants co-treated with HPE and IL-1 demonstrated marked reduction of MMP-1 (*p* = 0.001) and MMP-13 (*p* = 0.0023) expression compared to IL-1 alone. This effect was re-demonstrated in human cartilage with multiple MMP decreased. Reduced levels of GAG release occurred in HPE-treated cartilage explants in comparison to saline control (*p* = 0.004), with maintenance of structural architecture on histologic preparations.

**Conclusions:**

HPE may be a promising treatment for OA by inhibiting the metabolic process of ECM breakdown and preserving the structural integrity of the knee joint in comparison to saline control.

## 1 Introduction

Osteoarthritis (OA) often targets the knee, hip, and hand, with defined radiographic evidence of articular cartilage degeneration, subchondral sclerosis, joint space narrowing, and reactive osteophyte formation [[1], [2], [3]]. These pathologic changes contribute to pain and decreased mobility, severely limiting patient-perceived quality of life [4,5]. The overwhelming regularity of patients presenting for pain and disability necessitates a greater understanding of underlying mechanisms at play and exploration into new modalities for treatment and prevention of OA.

In the traditional context, OA is an irreparable degenerative disease, mediated by mechanical destruction and inflammation following chronic stress or injury. Recent research in the alterations of cellular homeostasis in OA has spurred interest in developing novel therapeutics beyond our current non-surgical options of lifestyle modifications; pain management of non-steroidal anti-inflammatories (NSAIDs), intra-articular injections of steroids, platelet-rich plasma, or hyaluronic acid (HA) [[6], [7], [8], [9]]. Surgical treatments include cartilage restoration through microfracture, osteochondral grafting, and total joint arthroplasty, although these invasive procedures may not be appropriate nor desirable for all patients [[10], [11], [12]].

Human birth tissues including amniotic membrane and amniotic fluid have long been used in therapeutic applications; their efficacy may be a result of the placental-derived growth factors and cytokines they contain [[13], [14], [15]]. Initial clinical studies of intra-articular injections of amniotic fluid preparations suggest that amniotic fluid can serve as an effective and durable therapeutic agent for post traumatic osteoarthritis (PTOA) and knee OA [13,[15], [16], [17], [18], [19], [20], [21]].

Human placental extracts (HPE) are acellular preparations of hydrolyzed full-term donor placental tissue, retaining the supraphysiologic levels of cytokines and growth factors found in native tissue with minimum manipulation [6,22]. A previous study by Kim et al. [23] examined the effect of an HPE on a monoiodoacetate (MIA)-induced OA rat model [24]. Kim’s group demonstrated their HPE’s efficacy in diminishing proteoglycan degradation, matrix metalloproteinase (MMP) activity, and histological changes in HPE treated groups [23]. In a separate study by Flannery et al. [21], the author derived a placental tissue biologic, PTP-001, that was similarly shown to inhibit MMP-13 production, promote synovial cell growth, and reduce the pain response in a rat OA model. To our knowledge, these are the sole studies demonstrating the relationship between HPEs and direct cartilage preservation.

To evaluate the protective effect of an HPE (PKTX-19) on articular cartilage in OA development, we examined the functional and histological outcomes at 1, 2 and 3 months following HPE treatment in a post-traumatic osteoarthritis (PTOA) rodent model. *In vitro* and *ex vivo* experiments were performed on healthy porcine articular as well as human total knee arthroplasty (TKA) cartilage explants to assess the effect of HPE on extracellular matrix (ECM) degradation. We hypothesize that this HPE would preserve cartilage integrity and mitigate OA progression by diminishing cartilage catabolism and restoring balance of homeostasis.

## 2 Materials and methods

### 2.1 Donor criteria and placental tissue preparation

Placental tissue and amniotic fluid were collected following informed consent obtained from 10 cesarean section delivery patients. Donor eligibility criteria were established in compliance with Good Tissue Practices promulgated by the FDA, the American Association of Tissue Banks Standards for Tissue Banking, and criteria of the local establishment’s Medical Director.

The placental tissue was prepared for use as described by Washburn [25]. Briefly, placental discswere grossly chopped into approximately 1 cm pieces and placed in Dulbecco’s phosphate buffered saline for washing and The tissues then further finely chopped in a laboratory blender in the final hydration solution and pooled. Cells were lysed, and the mixture was centrifuged to separate non-soluble debris. The final solution was freeze dried and terminally sterilized with 2MRad of gamma irradiation to generate PKTX-19.

### 2.2 Multiplex analysis

Freeze-dried samples were rehydrated in Dulbecco’s phosphate buffered saline to allow for total protein assay on PKTX-19 (Pierce™ Coomassie Protein Assay, ThermoFisher, MA). Concentrations of HPE were measured with a Bio-Plex Pro Human Chemokine panel in 10 samples of human placental extract. The plates were then read on a Bio-Plex MAGPIX Multiplex Reader at 525 and 635 nm wavelength (Bio-Rad CA).

### 2.3 Ex vivo *study: porcine explant glycosaminoglycan assay and histology*

To assess the safety and efficacy of PKTX-19 on attenuating glycosaminoglycan and matrix metalloproteinases (MMPs) release *ex vivo*, articular cartilage was shaved off in 3 mm thickness then collected by biopsy punch (6 mm diameter, McKesson, TX) from the medial tibial plateau of healthy 6-12 month-old pigs (*n* = 4) and cultured in Dulbecco’s Modified Eagle Medium supplemented with 1 % Penicillin-Streptomycin and Insulin-Transferrin-Selenium (ITS) (Sigma Aldrich, MO) for 48 h to reach equilibrium. To induce an arthritic phenotype, explants were stimulated with the pro-inflammatory cytokine IL-1α (10 ng/ml, Cell Signaling Technology, MA in the presence of PKTX-19 at low dose (LD) (3.75 mg/ml) or high dose (HD) (5.625 mg/ml) for another 48 h. After the induction period, conditioned media was collected, and glycosaminoglycan (GAG) content was measured using a dimethyl methylene blue assay (Bicolor Life Science, UK) then normalized with ds DNA released into media per explant weight. (Quant-iT™ PicoGreen™ dsDNA Assay Kit, Themo Fisher, MA).

For explant histology analysis, porcine cartilage proteoglycan degradation was assessed with Safranin-O staining. Hyaluronic acid (1:200 HA, Sigma-Aldrich, MO) deposition was measured using immunohistochemistry.

### 2.4 Western blot assay

**Fig. 1:**
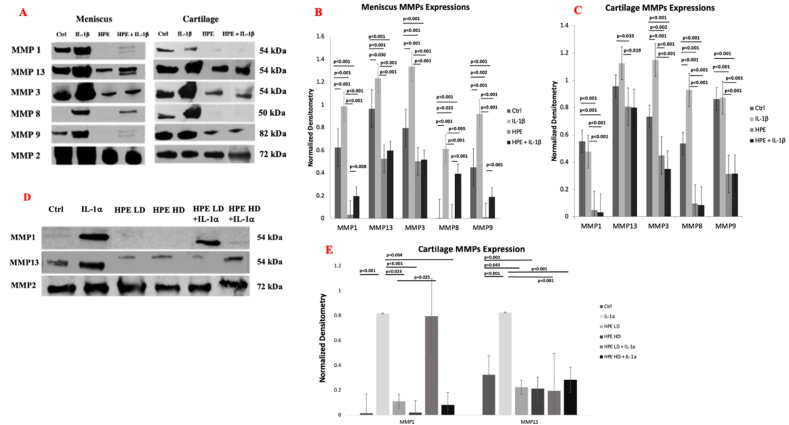
**(1A)** Representative human *ex vivo* cartilage and meniscus MMP-1, MMP-13, MMP-8 and MMP-9 detection within treatment groups at 48 h. MMP-2 was used as a loading control. IL-1β (10 ng/ml), HPE (5.625 mg/ml); Matrix metalloproteinase expression in human TKA meniscal **(1B)** and cartilage **(1C)** explants depicted as Western blot densitometry readings. **(1D)** Porcine *ex vivo* cartilage MMP-1 & MMP-13 within treatment groups at 48 h. IL-1α (10 ng/ml), HPE Low Dose (LD, 3.75 mg/ml), HPE High Dose (HD, 5.625 mg/ml). **(1E)** Porcine *ex vivo* cartilage explants MMP expression depicted as Western blot densitometry readings. Significance depicted as ∗ (*p* < 0.05) ∗∗ (*p* < 0.005).

Human cartilage explants were collected from TKA patients and approved by Wake Forest University School of Medicine Institutional Review Board. Cartilage was shaved off the superficial region of medial tibial plateau then biopsy punched to explants in 6 mm diameter and 3 mm thickness. The medial meniscus was cut transversely in 4 mm thickness then cultured directly in Dulbecco’s Modified Eagle Medium containing 10 % FBS and 1 % of antibiotics. The cartilage and meniscus were cultured in medium for 48 h before equilibrium then treated with either IL-1 β (10 ng/ml, Cell Signaling Technology, MA) or varying doses of PKTX-19 for another 48 h. MMP-1, MMP-3, MMP-8, MMP-9 and MMP-2 (Abcam, Cambridge, UK) expressions were examined in culture medium from human TKA explants (*n* = 4). Immunoblots for MMP-1 and MMP-13 (Abcam, Cambridge, UK) were assessed from porcine hyaline cartilage explants (*n* = 4). MMP-2 served as a loading control (Fig. 1).

### 2.5 In vivo *study: rodent PTOA model*

The *in vivo* experimental procedures were approved by the Wake Forest University School of Medicine Institutional Animal Care and Use Committee. 24 male and 23 female 3-month-old skeletally mature Lewis rats underwent destabilization of the medial meniscus (DMM) and anterior cruciate ligament (ACL) transection to induce post traumatic osteoarthritis (PTOA). Briefly, the animals were anesthetized with isoflurane, shaved and disinfected. A 4 mm anterior incision superior and medial to the tibial tubercle was made in the left medial parapatellar to expose the knee joint capsule. The patella was dislocated laterally, and the knee placed in full flexion followed by DMM and ACL transection. After surgery, the joint surface was washed with sterile saline solution, and both capsule and skin were closed using 5-0 vicryl suture. At 14 days post-surgery, each rat received an intra-articular injection in the joint with 0.10 mL of either sterile saline or PKTX-19 at a dose of 3.75 mg/ml. The saline cohort had six rats per collection period (*n* = 6 at 1,2 and 3 months, respectively). PKTX-19 cohort consisted of ten animals per month, apart from the last point having nine animals (*n* = 10 at 1 and 2 months, *n* = 9 at 3-months). Animals were tended to daily and multi-level pain regimen were administrated to address the post operative pain under the guidance of veterinarians. Gait analysis was performed at the end of each time point, followed by euthanasia of the animal and subsequent micro-CT imaging and histological preparation of the knee joints.

### 2.6 In-vivo *histological assessment*

#### 2.6.1 Osteoarthritis Histopathology Assessment system (OARSI) scoring

Harvested knee joints were placed in ethylenediaminetetraacetic acid (EDTA, Sigma-Aldrich, MA) based decalcifying solution for 14 days. Knee joints were processed and embedded with paraffin. Serial frontal sections were stained with Hematoxylin and Eosin (H&E) and Safranin O. The slides were then randomized and blindly evaluated microscopically using the OARSI scoring method for cartilage degradation [[26], [27], [28]].

### 2.7 Micro-CT analysis

The injured limb of eighteen rats was scanned by Bruker SkyScan microCT (Scanco Medical AG, Switzerland) with isotropic voxels of 10μm/side, at 70 kV with an intensity of 114 μA and a 1 mm aluminum filter. Rotation step and frame averaging were balanced to optimize scan time and image quality. Three-dimensional images were reconstructed using the NRecon Bruker software restricted to regions of interest. Knees were scanned at one- and two-months post PKTX-19 or saline intra-articular injection. (*n* = 6 for saline, *n* = 3 for PKTX-19 (3.75 mg/ml) per time point, respectively)

MicroCT data was then imported into Mimics Innovation Suite (v.18.0x64) and reoriented to the same coronal and sagittal plane for joint space analysis. For each joint, the narrowest cartilage-cartilage contact point between the femur and tibial plateau was first identified on sagittal imaging then corresponded on the coronal view for the joint space distance measurements. 10 consecutive slices were analyzed per sample and the average contact point distance was used for statistical analysis (Fig. 3A and B).

**Fig. 2:**
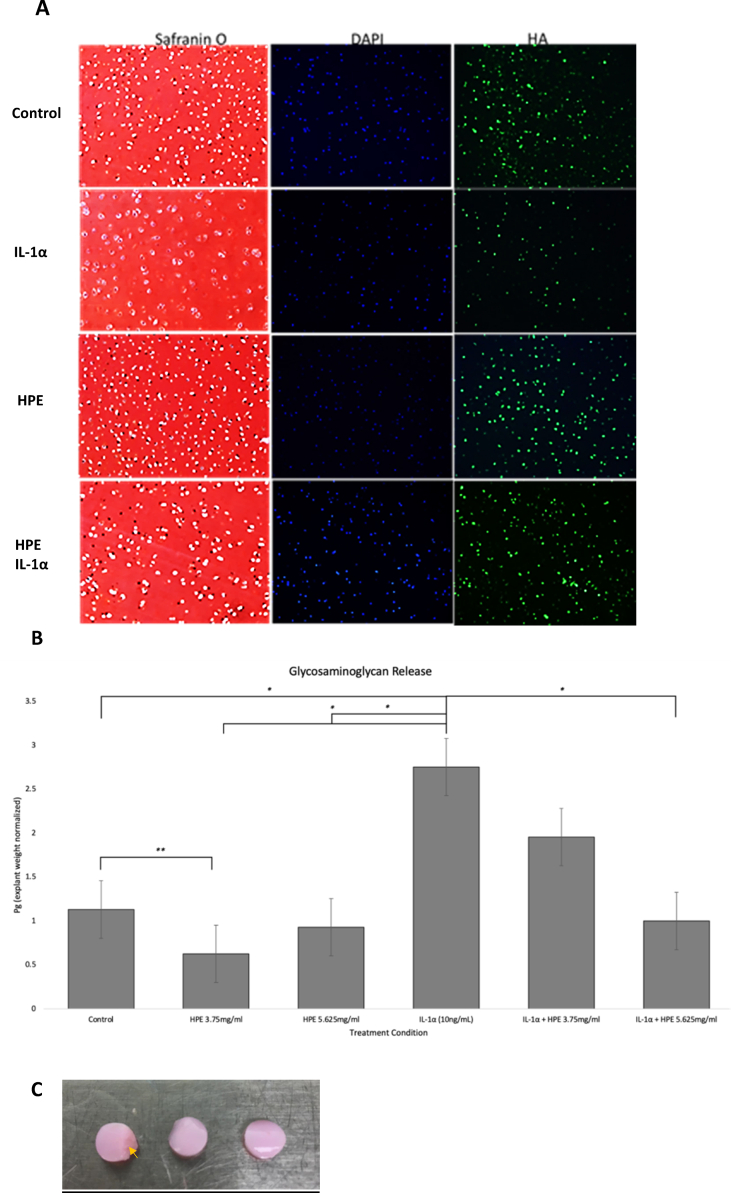
**(2A)** IL-1α (10 ng/ml) induced degenerative morphologic change and loss of chondrocytes in porcine articular cartilage explants after 48 h. HPE (5.625 mg/ml) rescued the cartilage degeneration and loss of HA deposition around the chondrocytes (magnification ×200); **(2B)** ECM degradation in porcine articular cartilage explants assessed via glycosaminoglycan (GAG) release at 48 h. GAG content normalized by explant weight (∗*p* < 0.05; ∗∗*p* < 0.005). (**2C**) Superficial zone of the cartilage explants (Arrowhead).

**Fig. 3:**
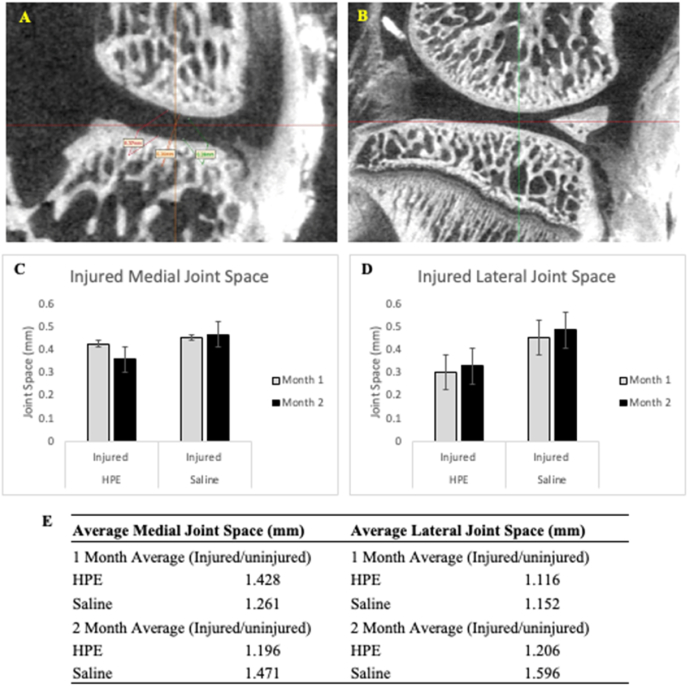
Coronal **(3A)** and sagittal **(3B)** view of rat knee femoral condyle and tibial plateau used to measure joint space distance at narrowest cartilage contact point; Average medial joint space **(3C)** and lateral joint space **(3D)** for injured limb in HPE and Saline cohorts; Joint space evaluation **(3E)**.

### 2.8 Gait analysis

Gait analysis was performed at baseline before surgery, and 1 month, 2 months and 3 months post either saline or PKTX-19 treatments using the DigiGait System (Mouse Specifics, MA). Animal weight was monitored at each time point throughout the study. The DigiGait system runs an automated gait analysis by capturing the ventral plane of the rat as it walks on a translucent treadmill belt and images the underside of the paws continuously with a high-speed digital video camera (176 frames per second). Digital paw prints are generated and translated to dynamic gait signal. The captured gait was then analyzed using DigiGait imaging analysis software. The gait parameters that were used for analysis have previously been validated [29].

### 2.9 Statistical methods

*In vivo* MicroCT, gait analysis and OARSI results were analyzed using one-way analysis of variance for comparisons between treatment conditions at all measured endpoints. Post-hoc Bonferroni correction was used to adjust for multiple comparisons. If data was non-parametric Welch’s test was used to assess for significance – this was only the case in the one-month lateral joint space MicroCT analysis. Student’s t-test was performed for between group comparisons for *in vitro* and *ex vivo* analysis at 48 h. Statistical significance was defined as a *p*-value <0.05. Statistical tests were run using IBM SPSS Vs. 29.

## 3 Results

### 3.1 Characterization of human placental extract

Using a Bio-Plex 40 Multiplex assay, all 40 components were identified in variable concentrations within the human placental extract samples. It was found that PKTX-19 contains numerous trophic growth factors including basic fibroblast growth factor, platelet-derived growth factor-beta (PDGF-β) and stromal cell-derived factor 1. A full representation of the composition of PKTX-19 is described in Table 1.

**Table 1:** Analyte concentrations in human placental extract.

	Mean Concentration (pg/mL)	Standard Error	% CV
6Ckine/CCL21	349.8	38.4	62.1
BCA-1/CXCL13	25.5	2.8	62.8
CTACK/CCL27	27.6	3.4	69
ENA-78/CXCL5	800.8	131.4	54.4
Eotaxin/CCL11	27.7	3.2	65.4
Eotaxin-2/CCL24	141.9	35.5	74.9
Eotaxin-3/CCL26	11.6	1.5	73.1
Fractalkine/CX3CL1	261.2	29.9	64.7
GCP-2/CXCL6	154.7	19.9	72.7
GM-CSF	30.8	3.1	64.4
GRO-a/CXCL1	1065.8	131.2	69.6
GRO-b/CXCL2	451.5	53.9	67.6
I-309/CCL1	69.6	7.1	57.7
IL-10	21.2	2.4	64.9
IL-16	436	52.5	68.1
IL-1beta	27.5	3.7	75.5
IL-2	7.7	0.8	59.1
IL-4	9	1	59.6
IL-6	230	36.1	88.8
IL-8/CXCL8	9535.7	2289.4	135.8
INF-gamma	85.9	11.1	133.5
IP-10/CXCL10	241.1	24.6	57.7
I-TAC/CXCL11	38	4.4	66.2
MCP-1/CCL2	684.8	93.8	77.5
MCP-2/CCL8	188.8	21.3	63.9
MCP-3/CCL7	88.6	9.6	61.5
MCP-4/CCL13	101	14.3	80
MDC/CCL22	167.3	20.5	69.3
MIF	154,285.5	17,528.3	64.3
MIG/CXCL9	209.5	22.1	59.7
MIP-1alpha/CCL3	98.4	9.7	55.9
MIP-1delta/CCL15	210.2	20.1	54.2
MIP-3alpha/CCL20	17.9	3.9	124.9
MIP-3beta/CCL19	55.8	6.3	64.3
MPIF-1/CCL23	50	7.3	83.1
SCYB16/CXCL16	35.9	3.1	48.4
SDF-1 alpha-beta/CXCL12	269.2	25.9	54.4
TARC/CCL17	20.4	3	73.6
TEC/CCL25	1012.3	81.4	45.5
TNF-alpha	39.5	5.1	73.2

### 3.2 Ex-vivo *human and porcine explant*

#### 3.2.1 Human TKA explant MMP expression

Human osteoarthritic cartilage explants expressed all MMP-1, MMP-13, MMP-3, MMP-8 and MMP-9 at equilibrium before treatments. IL-1β significantly further induced MMP-3 and MMP-8 expression compared to the control at 48 h. PKTX-19 (5.625 mg/ml) decreased all MMP-1, MMP-13, MMP-3, MMP-8 and MMP-9 expression remarkably compared to control explants alone (*p* < 0.01). This effect was also observed in the explants co-treated with IL-1β, indicating the prevention of early ECM breakdown with PKTX-19 in the presence of pro-inflammatory mediator IL-1β (*p* < 0.01, Fig. 1A and C).

PKTX-19 treated meniscus demonstrated marked reduction across all MMP expressions when compared to the OA control meniscus explant (*p* < 0.01, Fig. 1A and B). Furthermore, this relationship was maintained in the IL-1β co-treated explants, showing significant decrease in all MMP expressions with PKTX-19 treatment (*p* < 0.01, Fig. 1A and B).

#### 3.2.2 Porcine cartilage MMP-1 and MMP-13 analysis

IL-1α significantly increased MMP-1 expression in comparison to healthy porcine articular cartilage control, but this effect was only inhibited when co-treated with PKTX-19 HD (5.625 mg/ml, *p* = 0.004) (Fig. 1D and E).

IL-1α also significantly promoted MMP-13 release from explants compared to healthy control (*p* = 0.001) and IL-1α induced MMP-13 expression was decreased when co-treated with PKTX-19 compared to the ones exposed to IL-1α alone. (*p* < 0.01 LD and HD respectively). PKTX-19 LD (3.75 mg/ml) and HD (5.625 mg/ml) both exhibited a significant decrease in MMP-13 expression in comparison to control (*p* < 0.01). (Fig. 1D and E).

#### 3.2.3 Glycosaminoglycan (GAG) release and explant histology

Histological analysis with Safranin-O staining of porcine explant articular cartilage after 48-h of treatment demonstrated IL-1α induced loss of superficial chondrocytes, cellular hypertrophy and pericellular proteoglycan breakdown. Loss of HA in ECM was also observed with in IL-1α treated explants (Fig. 2A). There was considerable rescue of cartilage structure and HA deposition surrounding chondrocytes in the PKTX-19 HD cohort.

GAG release was diminished in the PKTX-19 LD (3.75 mg/ml) group in comparison to control (*p* = 0.004) but did not show a significant difference in the PKTX-19 HD (5.625 mg/ml) group in comparison to control (*p* = 0.414) (Fig. 2B). Both PKTX-19 LD and HD decreased GAG release in comparison to IL-1α treatment alone in a statistically significant manner (*p* = <0.05) with control also showing a significantly decreased GAG release when compared to IL-1α (*p* = *0.001).*

In the co-treated PKTX-19 LD and HD with IL-1α groups, only PKTX-19 HD co-treated with IL-1α decreased GAG release in comparison to IL-1α in a significantly (*p* = *<*0.05).

### 3.3 In vivo *rodent results*

#### 3.3.1 Gait analysis

Comparing PKTX-19 to saline injection at 1 month yielded significant differences in 5 of the measured parameters (Table 2, Table 3). These include Propel (*p* = 0.003), Stance (*p* = 0.016), Stride (*p* = 0.007), Stride Frequency (*p* = 0.015), and Stride Length (*p* = 0.008) (Table 3). None of these variables maintained significance at the later points of two or three months. Only Swing-Stance (*p* = 0.009), and Swing (*p* = 0.043) both showed a significant difference between PKTX-19 and saline at the later three-month interval with swing-stance being greater in the PKTX-19 cohort (Table 3).

**Table 2:** Gait analysis: Mean values of injured limb.

	Human Placental Extract (HPE)				Saline			
	Baseline	1 Month	2 Months	3 Months	Baseline	1 Month	2 Months	3 Months
Absolute paw angle (°)	9.297 (4.55)	13.709 (4.97)	17.867 (5.38)	15.514 (2.19)	10.940 (2.85)	15.725 (3.75)	17.633 (2.47)	10.780 (7.70)
Brake (s)	0.035 (0.01)	0.048 (0.03)	0.040 (0.01)	0.057 (0.02)	0.050 (0.03)	0.054 (0.01)	0.042 (0.01)	0.039 (0.02)
Paw angle variability (°)	6.276 (5.41)	3.518 (1.79)	4.489 (2.59)	2.714 (1.27)	3.680 (1.89)	5.300 (5.08)	4.467 (2.21)	4.940 (1.56)
Propel (s)	0.295 (0.03)	0.323 (0.04)	0.316 (0.05)	0.314 (0.04)	0.273 (0.05)	0.282 (0.04)	0.316 (0.01)	0.317 (0.03)
Stance (s)	0.330 (0.03)	0.371 (0.04)	0.356 (0.05)	0.371 (0.04)	0.323 (0.04)	0.336 (0.03)	0.358 (0.01)	0.356 (0.04)
Swing-stance (s)	2.638 (0.40)	2.682 (0.35)	2.611 (0.54)	2.957 (0.67)	2.660 (0.51)	2.800 (0.61)	2.767 (0.78)	2.540 (0.30)
Stride (s)	0.457 (0.03)	0.510 (0.05)	0.495 (0.05)	0.501 (0.05)	0.446 (0.04)	0.458 (0.04)	0.494 (0.03)	0.500 (0.07)
Stride frequency (steps/s)	2.230 (0.19)	2.027 (0.24)	2.089 (0.20)	2.014 (0.29)	2.313 (0.16)	2.225 (0.21)	2.067 (0.15)	2.020 (0.30)
Stride length (cm)	11.424 (0.83)	12.745 (1.23)	12.389 (1.22)	12.514 (1.27)	11.160 (1.02)	11.475 (0.87)	12.333 (0.81)	12.500 (1.74)
Swing (s)	0.127 (0.02)	0.139 (0.02)	0.139 (0.02)	0.130 (0.03)	0.123 (0.01)	0.122 (0.02)	0.136 (0.04)	0.144 (0.03)

Values reported as Mean (Std Dev) for each parameter.

**Table 3:** p-Values Indicating Significant Longitudinal Gait Differences Within Cohorts.

Human Placental Extract (HPE)					
Baseline vs. 1-Month		Baseline vs. 2-Months		Baseline vs. 3-Months	
Parameter	*p*-value	Parameter	*p*-value	Parameter	*p*-value
*Brake*	0.041	*Absolute Paw Angle*	<0.001	*Absolute Paw Angle*	0.041
*Propel*	0.011	*Stride*	0.028	*Propel*	0.013
*Stance*	<0.001	*Stride Frequency*	0.031	*Stance*	<0.001
*Stride*	<0.001	*Stride Length*	0.026	*Swing-Stance*	0.016
*Stride Frequency*	<0.001			*Stride*	0.002
*Stride Length*	<0.001			*Stride Frequency*	0.007
*Swing*	0.006			*Stride Length*	0.002

**Saline**					

Baseline vs. 1-month		Baseline vs. 2-months		Baseline vs. 3-months	

Parameter	*p*-value	Parameter	*p*-value	Parameter	*p*-value

*Absolute Paw Angle*	0.032	*Propel*	0.039	*Propel*	0.020
				*Stride*	0.004
				*Stride Frequency*	0.013
				*Stride Length*	0.003

**Human placental extract (HPE) vs saline**					

HPE vs. Saline at 1-month		HPE vs. Saline at 2-months		HPE vs. Saline at 3-months	

Parameter	*p*-value	Parameter	*p*-value	Parameter	*p*-value

*Propel*	0.003			*Swing-Stance*	0.009
*Stance*	0.016			*Swing*	0.043
*Stride*	0.007				
*Stride Frequency*	0.015				
*Stride Length*	0.008				

When assessing the longitudinal changes within the PKTX-19-injection cohort, 7 of the recorded variables were significantly different between baseline and 1-month measurements (Table 2, Table 3). Of these, Stride, Stride Frequency, and Stride length were significantly different in comparison to baseline at all measured time points (Table 3). At the 3-month time point, 7 parameters were again significantly different from baseline, although the individual variables were different from the earlier one-month measurements. The longitudinal outcomes of the saline cohort yielded less significant findings, with a single parameter (absolute paw angle) being statistically significant against baseline at 1 month, and increasing to four parameters (propel, stride, stride frequency, stride length) at 3-month time point (Table 3). No weight differences were found between any time points in either PKTX-19 or saline group.

#### 3.3.2 MicroCT outcomes

The average medial joint space for 1 month was 0.425 mm in the PKTX-19 cohort and 0.450 mm in the saline cohort (Fig. 3C). Average medial joint space at 2 months was 0.356 mm for the PKTX-19 cohort and 0.465 mm in the saline cohort. Average lateral joint space at 1 month was 0.301 mm in the PKTX-19 cohort and 0.451 mm in the saline cohort (Fig. 3D). At 2 months, the average lateral joint space was 0.328 mm for the PKTX-19 cohort and 0.486 mm in the saline cohort. There was no statistical significance between PKTX-19 and saline group at either medial or lateral side at any time point after normalizing the injured limb joint space to the contralateral uninjured one (Fig. 3E).

#### 3.3.3 Osteoarthritis Histopathology Assessment System

**Fig. 4:**
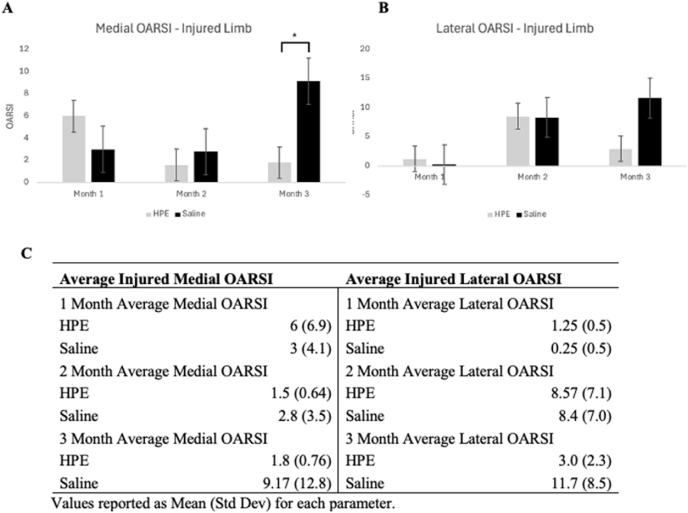
Average medial **(4A)** and lateral **(4B)** OARSI score for injured limb in HPE and Saline cohort. Mean values displayed for each treatment cohort at specific time interval. Statistical significance noted at 3 months between Saline and HPE cohort (*p* < 0.05); **(4C)** Histological OARSI Scoring Outcomes.

**Fig. 5:**
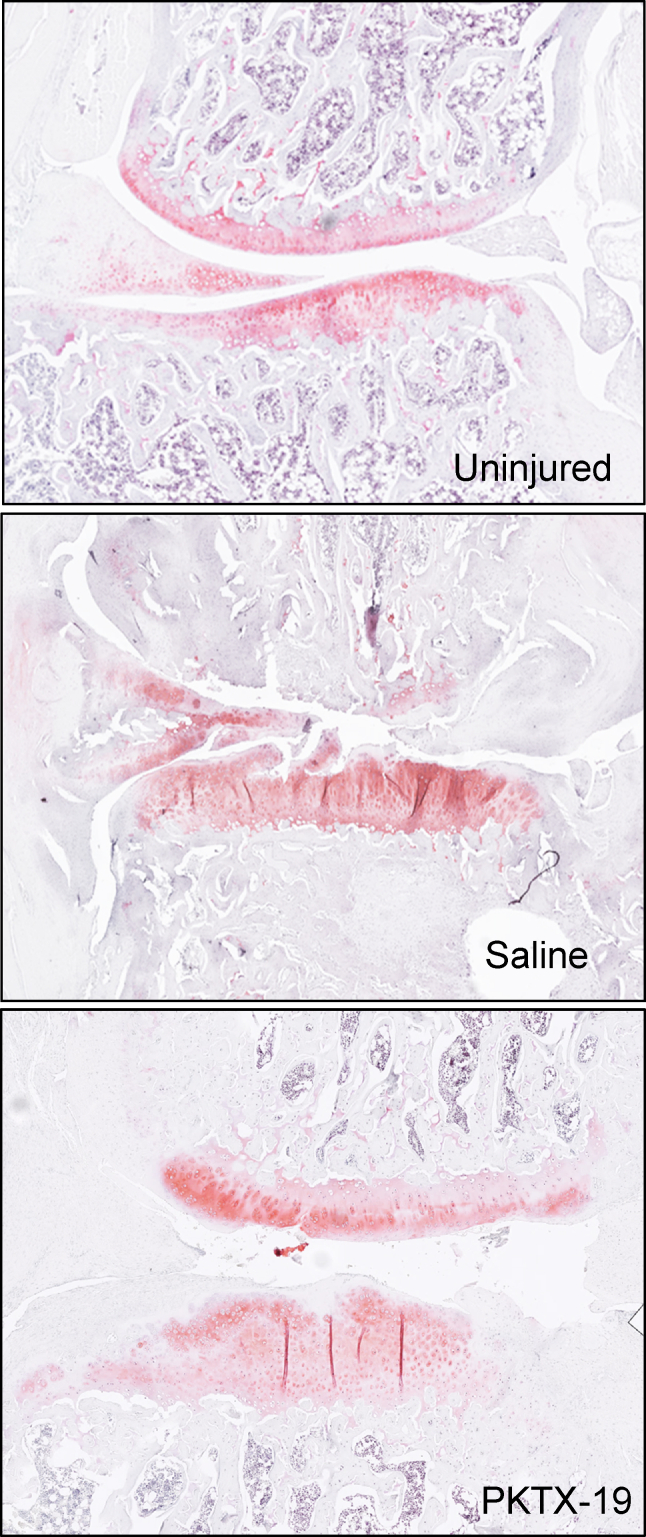
Representative Safranin-O staining of the medial compartment at 3-months in contralateral uninjured (top), saline control group **(middle)** and PKTX-19 treatment group **(bottom)**.

Lateral and medial OARSI scores both increased over time in the saline cohort (Fig. 4A and B). The scores in the PKTX-19 cohort showed a significant improvement at 2 months (1.58) in comparison to 1 month (6.0) and then a slight regression at 3 months in the medial joint space of the injured limb (1.8) (Fig. 4, Fig. 5). On the lateral side of the same limb, there was an increase in the OARSI score from 1 month (1.25) to 2 months (8.57) then subsided at 3 months (3.0). The only statistical significance was between the PKTX-19 and Saline cohort of the medial joint space in the injured limb at 3 months (Fig. 4A) (*p* = *<0.05)*. Mann-Whitney *U* test was performed on OARSI scores due to non-normal distribution of the data.

## 4 Discussion

To date, the number of effective treatments for knee OA are limited [30]. Most treatment algorithms utilize a ladder progressing from rest, ice compression and elevation to NSAIDs, corticosteroid injections and eventually joint replacement [7,31]. The absence of potent therapeutics addressing the underlying progression of OA limits options for patients with this debilitating disease. The potential for PKTX-19 as a therapeutic is promising in that it targets cellular metabolism. Studies characterizing the composition of PKTX-19 while examining its role in wound healing found high levels of transforming growth factor-beta, fibroblast growth factor, epidermal growth factor, platelet-derived growth factor, insulin-like growth factor 1, and vascular endothelial growth factor - many growth factors that promote healing and regeneration [32,33]. This makes PKTX-19 an exciting candidate for application in osteoarthritis, as its administration will provide cytokines and growth factors theorized to be deficient by inhibited cellular pathways in senescent chondrocytes [34].

We first examined the safety and effect of PKTX-19 using the *ex vivo* healthy porcine cartilage explants. PKTX-19 treated cartilage showed improved structural integrity on histology of the chondrocytes and surrounding ECM. The first degenerative change in healthy articular cartilage after acute induction of IL-1α for 48 h was found in the matrix and chondrocytes of the superficial zone. These changes consisted of loss of superficial chondrocytes and ECM with the presence of empty lacunae, while the remaining chondrocytes transformed to the hypertrophic phenotype, resembling the cells from deeper zones. PKTX-19 treatment dramatically reversed the transformation of chondrocytes and the breakdown of ECM in response to the acute inflammatory cue, suggesting the effective inhibition of inflammatory cascades that leads to accelerated catabolism in the acute stage of OA progression.

The measured GAG release in the porcine explants confirms rescue of matrix degradation with PKTX-19, which aligns with the restored proteoglycan content as measured by Safranin-O staining. This finding shows that the ECM was more consistently preserved with PKTX-19 treatment in comparison to the explants treated with IL-1α, demonstrating that the catabolic process during the acute inflammatory stage of OA was mitigated. The improvement in structural integrity on histological analysis was also corroborated on analysis of MMP1 and MMP13 biomarkers in the explant media as both decreased with PKTX-19 treatment, suggesting PKTX-19 was involved in MMP mediated matrix breakdown when IL-1α was present.

We further assessed the effects of PKTX-19 on the explants collected from TKA patients under chronic OA condition. PKTX-19 consistently reduced multiple MMPs compared to untreated control and IL-1β-stressed explants. This is especially intriguing as high levels of multiple MMPs which are present in advanced-stage osteoarthritic explants can also be significantly reversed, suggesting that PKTX-19 has the potential to mitigate chronic catabolism in primary OA as well. A similar study by O’Brien et al. used a coculture system of cartilage and synovium from TKA patients and found that amniotic fluid had anti-inflammatory effects by decreasing ADAMTS-5 and TIMP-1 gene expression when co-treated with IL-1β for 72 hours [35].

Another study by Flannery et al. demonstrated that PTP-001, a human placental tissue-derived biologic, can significantly reduce MMP-13 in OA chondrocyte-culture media, suggesting protection against cartilage breakdown [21]. These findings were in line with our observation that PKTX-19 counteracted inflammation triggered multiple MMP release in both healthy porcine and OA patient cartilage explants.

We also explored the effect of a one-time injection of PKTX-19 using a PTOA rat model. We observed different patterns of PTOA cartilage progression between PKTX-19 and saline treated groups in histology. On the medial injured side of joint, PKTX-19 treated group showed a continuous decline in scores from 1 month to 3 months whereas the saline control had the opposite inclining/worsening scores over time. This trend was also found on the lateral side with scores starting to climb from 1 to 2 months in both groups, probably due to the shift of load bearing following the medial side injury, then PKTX-19 depicted the cartilage recovery from 2 to 3 months whereas the score of the control group kept increasing. The improvement in OARSI score on both medial and lateral scores in PKTX-19 cohort demonstrates better regeneration of the articular cartilage at 3 months after DMM and ACLT injury, showing beneficial effects in promoting cartilage restoration in comparison to a continuous deterioration of cartilage breakdown in the saline group. Interestingly, in the present study PKTX-19 didn’t demonstrate a superior protective effect on the cartilage at around 3–4 weeks post treatment as reported by Flannery et al., where PTOA was conducted by DMM alone then treated by a placenta derived biologic, PTP-001 [21]. Rather, we found a more prominent cartilage recovery at later time in PKTX-19 treated animals, probably due to the varying severity of the PTOA induction (DMM vs. DMM + ACLT) and the duration of inflammatory state in the joint.

MicroCT results were in discordance to our expected findings. To our surprise, PKTX-19 didn’t preserve joint space compared to saline controls at either 1- or 2-months post intervention as found in histology (Fig. 3E). However, this finding was not statistically significant. The discrepancy between micro-CT and histological results could be due to several reasons. First, to best evaluate the treatment effects, we chose to use DMM in combination with ACL transection to induce PTOA, which creates a severe PTOA model to counteract rodents’ spontaneous healing. The joint could have been so severely damaged after the surgery that despite restoration of cartilage on histology, there was already sufficient subchondral bone loss and soft tissue compromise due to the inflammatory milieu, which led to the space between the tibial plateau and the femoral condyle permanently altered. Second, PKTX-19 showed significance in improving the cartilage restoration (lower OARSI score) more effectively at later point (3 months) post treatment. Unfortunately, we didn’t have enough animal numbers to analyze for both micro-CT and histology at 3-month point and we speculated that it is highly possible that micro CT would reflect the similar time course of joint recovery as shown in OARSI score. Third, it is entirely likely that the number of rats used for this study was insufficient. Being a pilot study, we postulate that to be properly powered, more rats would be needed to confirm our findings on imaging and histology.

For gait analysis, there was early variability with no consistent results between the PKTX-19 and saline cohorts that improved over time. Of note, stride length and stance improved for the PKTX-19 cohort by month 3 significantly whereas for the saline cohort there was no statistically significant difference and only worsening stance time (shorter time) between month 2 and 3. Stance time is an excellent surrogate for pain, in that if animals have more knee pain, they are less likely to put standing pressure on that knee. The opposite holds true in that if there is improvement in pain, as in the case with PKTX-19 treatment, the rodent will begin to spend more time on their injured limb.

In conclusion, PKTX-19 proved to be safe and had the potential to mitigate the progression of PTOA across *in vitro*, *ex vivo* and *in vivo* analysis. Both the metabolic process of ECM breakdown was inhibited and the structural integrity of the joint was improved with PKTX-19.

### 4.1 Limitations

The primary limitation related to this study is the number of rodents due to the nature of pilot study. Of the available literature on the topic, most published with smaller samples sizes ranging from 5 to 18 animals [14,[17], [18], [19], [20], [21],^23^]. This challenge was particularly evident in the analysis of joint space, The remaining significant findings across other measurements (GAG release, MMPs analysis, histologic OARSI scores and gait analysis) that all point to significant differences between PKTX-19 and saline control with improvement in structural and functional outcomes in PKTX-19 group. The secondary limitation is that PKTX-19 was administrated as one single injection at 2 weeks post-surgery and no positive control such as corticosteroid was included for treatment effects comparison. Future studies are still warranted to investigate the crucial perspectives for using PKTX-19 as an OA therapeutic: the optimal time and frequency of administration; the long-term outcomes of OA joints; and the underlying mechanisms of PKTX-19 as a potential biologic in treating OA.

## Author contributions

Conception and design: XM, TS.

Development of methodology:

Acquisition of data (provided animals, acquired and managed patients, provided facilities etc.): XM, ST, JC, SW, JS.

Analysis and interpretation of data (e.g. statistical analysis, biostatistics, computational analysis): XM, MB, MG, KH, AO, ST, SW.

Writing, review, and/or revision of the manuscript: XM, MB, MG, TS.

Administrative, technical, or material support (i.e., reporting or organizing data, constructing databases): XM.

Study Supersion: XM.

## Conflicts of interests

Author ST is a shareholder in Plakous Therapeutics Inc. Author SW serves as the founder, board chair, and shareholder of Plakous Therapeutics Inc, and holds patents for Acellular Placental Preparations (USPTO). Author JS is on the advisory board and serves as a consultant for Smith and Nephew.
